# Development and Characterization of Embroidery-Based Textile Electrodes for Surface EMG Detection

**DOI:** 10.3390/s22134746

**Published:** 2022-06-23

**Authors:** Hyelim Kim, Siyeon Kim, Daeyoung Lim, Wonyoung Jeong

**Affiliations:** 1 Material and Component Convergence R&D Department, Korea Institute of Industrial Technology (KITECH), Ansan 15588, Korea; hyelim1221@kitech.re.kr (H.K.); zoro1967@kitech.re.kr (D.L.); 2 Reliability Assesment Center, FITI Testing and Research Institute, Seoul 07791, Korea; siyeonkim@fitiglobal.com

**Keywords:** textile electrode, embroidery, surface electromyography, bio-signal, smart clothing

## Abstract

The interest in wearable devices has expanded to measurement devices for building IoT-based mobile healthcare systems and sensing bio-signal data through clothing. Surface electromyography, called sEMG, is one of the most popular bio-signals that can be applied to health monitoring systems. In general, gel-based (Ag/AgCl) electrodes are mainly used, but there are problems, such as skin irritation due to long-time wearing, deterioration of adhesion to the skin due to moisture or sweat, and low applicability to clothes. Hence, research on dry electrodes as a replacement is increasing. Accordingly, in this study, a textile-based electrode was produced with a range of electrode shapes, and areas were embroidered with conductive yarn using an embroidery technique in the clothing manufacturing process. The electrode was applied to EMG smart clothing for fitness, and the EMG signal detection performance was analyzed. The electrode shape was manufactured using the circle and wave type. The wave-type electrode was more morphologically stable than the circle-type electrode by up to 30% strain, and the electrode shape was maintained as the embroidered area increased. Skin-electrode impedance analysis confirmed that the embroidered area with conductive yarn affected the skin contact area, and the impedance decreased with increasing area. For sEMG performance analysis, the rectus femoris was selected as a target muscle, and the sEMG parameters were analyzed. The wave-type sample showed higher EMG signal strength than the circle-type. In particular, the electrode with three lines showed better performance than the fill-type electrode. These performances operated without noise, even with a commercial device. Therefore, it is expected to be applicable to the manufacture of electromyography smart clothing based on embroidered electrodes in the future.

## 1. Introduction

Research and development of wearable technology and devices have expanded rapidly to health, medical care, and convenience improvement. A wearable is defined as any technology that benefits from the human body as a platform. A wearable device is a smart device worn on the body for information and communication that can exchange continuously in real-time. Information can include the user’s biometric information, such as movement, heartbeat, respiration, and surrounding environment information (e.g., temperature, humidity, and harmful factors) [1,2,3,4,5,6]. Wearable technologies can be versatile and meet system requirements across domains because of the various sensors and actuators embedded in wearable devices. These devices can include smartwatches or smart clothing in terms of portability, human compatibility, and user interface because they are used in close contact with the human body [7,8,9,10]. The application of electronic devices for smart clothing for daily life is evolving from on-cloth to in-cloth.

The interest in wearable devices is increasing as a measurement device for building ICT (information and communications technology)- and IoT (Internet of Things)-based mobile healthcare systems. This technology has the advantage of acquiring real-time bio-signal data of the wearer through wireless communication with various sensors built into the wearable device. Accordingly, many studies on sensing bio-signal data through clothing are being conducted. Surface electromyography (sEMG) is a bio-signal and a valuable tool in biofeedback, sports medicine, and movement analysis [11]. sEMG can collect a large amount of information, such as the occurrence of muscle contraction and relaxation and the magnitude of muscle forces. Hence, it is being commercialized in occupational and sports medicine and smart wearable systems. On the other hand, smart wearable systems are limited by high prices and aesthetical dissatisfaction, comfort, and ease of use. Accordingly, smart clothing is being developed and studied to produce the same comfort as conventional clothing [12,13].

One of the significant issues for overcoming the limitation of smart clothing for EMG measurement is the electrode part. The electrode generally used to obtain a bio-signal, such as sEMG from a healthcare system, is an Ag/AgCl hydrogel electrode called a disposable electrode. There are several problems with disposable electrodes. Skin irritation occurs when used for a long time, and unreliable signals are sent when dry. In addition, because it is very sticky, it cannot be applied to clothes that need to be used for a long time. Moreover, even if it can be used multiple times, it is difficult to apply to a smart wearable system because the durability against adhesion is low. Thus, many studies have been conducted on dry electrodes to replace the wet electrodes used in the past [14,15,16].

Textile-based electrodes, a type of dry electrode, are being developed by fabricating fabrics, such as knitting, weaving, and embroidery, using metal wire or conductive yarn, or coating or printing on various substrate materials [15,16,17,18,19]. In this case, the inner electrode of the clothing must be in contact with the skin to collect bio-signals, and the outer electrode must be designed to connect to a device capable of collecting electromyography. Textile-based electrodes are built into clothes and are able to manufactured to be similar to clothes worn in daily life. Thus, it is easy to put on and takes off, and there is almost no foreign body feeling to the electrodes, so the wearing comfort is improved. A representative method for fabricating textile-based electrodes involves manufacturing a woven or knitted fabric using conductive yarn [20,21,22] or a method of manufacturing by screen-printing on fabric using a conductive ink or a composite [23]. Among them, embroidery is used to produce textile-type electrodes. An embroidery electrode can collect internal and external signals from inside and outside based on the fabric. In addition, conductive patterns can be made on the finished textile surface. Furthermore, the base material can be laid in all directions rather than pre-defined ones [24,25,26]. In addition, textile-based embroidery electrodes can be redesigned easily to comply with different users’ needs because they can be designed by software. Therefore, it could enhance the aesthetics of smart clothing and skin-electrode contact.

Several studies on the collection performance of the textile-type electrode are continuously being carried out. Pitou et al. (2020) examined the EMG detecting performance using hand-made or basic embroidery machines but reported restrictions on the esthetics and mass production of smart clothing [26]. Goncu-Berk and Tuna (2021) analyzed the EMG signal detecting performance using embroidery electrodes [27]. A comparison was made between conventional Ag/AgCl hydrogel electrodes and embroidery electrodes. A sleeve was manufactured as a sample and compared with a raglan sleeve and a set-in sleeve. As a result of the experiment, it was reported that when the sleeve with the textile-based embroidery electrode was worn, the EMG signal was detected more stably because it fit the human body well. In a previous study [28], an electrode was fabricated using a conductive sheet to replace the disposable electrode. A leg sleeve was fabricated to place the electrode on the rectus femoris. The EMG signal detecting performance was then evaluated according to the electrode size and pattern reduction rate (PRR) rate. As a result, when the electrode size was 25 mm, the EMG signal could be collected without noise even if the PRR was changed. Nevertheless, the limitation of using a metal snap to collect both internal and external signals was confirmed because the conductive sheet was attached to only one side of the fabric. Therefore, this study manufactured textile-based electrodes for sEMG detection in EMG module-combined smart clothing. Two types of electrodes were designed using embroidery techniques, and their sEMG performance was investigated. First, the embroidery electrodes were designed in a circle-type and wave-type, considering elasticity when wearing clothes, and those were designed according to three different densities for each shape. Bipolar electrodes with a constant inner-electrode distance were placed, and a leg sleeve type was fabricated to evaluate the sEMG performance of the rectus femoris muscle. Finally, for performance evaluation according to electrode shape and density, DIC analysis through a tensile test, skin-electrode impedance, and sEMG signal test was evaluated using the biopac and wearable devices. In addition, to confirm durability against wear during actual use and applicability, it was analyzed by measuring the change in resistance by the number of abrasions and signal sensing performance evaluations. 

## 2. Experimental

### 2.1. Materials

**Figure 1:**
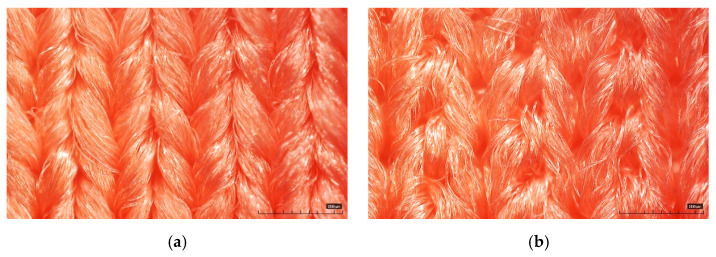
The morphology of substrate fabric used in this study: (**a**) Face side; (**b**) back side.

The substrate fabric used to manufacture the embroidery electrode was a polyester-based elastic fabric selected for application as smart sportswear. The fabric was composed of 88% polyester and 12% spandex, and the weight and thickness were 540 g/yd and 1.0 mm, respectively. The used substrate fabric was a double weave (wale × course, 128 × 88/5 cm). The face of the fabric was composed of a flat knitted fabric, and the back side was a double-sided knitted fabric (Figure 1). The designed electrodes were manufactured using a technical embroidery machine (SGVA 0109-825, ZSK Stickmaschinen GmbH, Krefeld, Germany). The needle used for the embroidery machine was a No. 14 with 90 Nm. At this time, the conductive yarn from the AMANN group (Silver-tech, AMANN group, Bönnigheim, Germany), which has a 22 Tex and resistivity of 530 Ω/m, was used for the upper and lower yarns.

### 2.2. Development of Prototype

#### 2.2.1. Design and Preparation of Embroidery Electrodes

Table 1 lists the sample codes and each embroidery electrode image. In this study, two shapes (circle-type (C) and wave-type (W)) with different numbers of lines were selected to confirm the sEMG signal by the design factors to compare the change according to the electrode shape and density. The samples were designed using the EPC_win embroidery design program (ZSK Stickmaschinen GmbH). The electrode diameter was set to 20 mm, and the embroidery parameters of stitch length and distance were applied as 2.0 mm and 0.5 mm, respectively. At that time, two identical electrodes were designed with a distance between their centers (IED, inter-electrode distance) equal to 40 mm according to a previous study [28]. Subsequently, each sample was embroidered on substrate fabric using silver-based conductive embroidery yarn and a technical embroidery machine.

#### 2.2.2. Preparation of Leg Sleeves Embedded the Embroidery Electrodes

**Figure 2:**
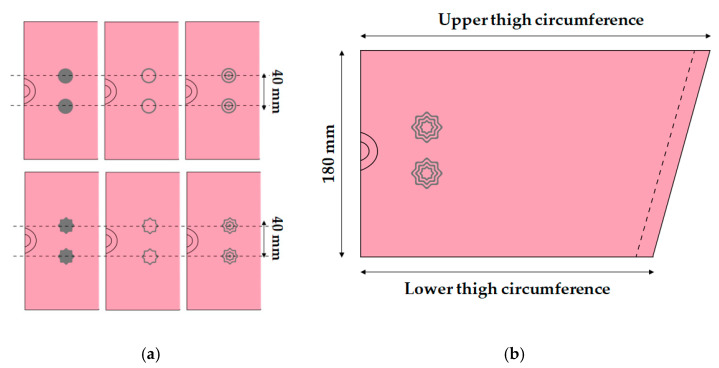
Scheme of fabrication of leg sleeves with embedded embroidery electrodes: (**a**) IED (inter-electrode distance) and (**b**) pattern image of sample.

The leg sleeves to measure the sEMG signals were fabricated by applying the measurement position to the rectus femoris muscle. The electrode position was selected so that two embroidery electrodes could be located at the corresponding position. The current study was perform on a subject for sEMG measurements (sex = female, age = 31, height = 175 cm, body weight = 60 kg). According to the subject’s condition, the width was 180 mm based on the electrode part, and the circumferences of the upper and lower thighs corresponding to the position were measured. According to the previous study [28], a 30% pattern reduction rate (PRR) was applied, considering the elasticity of the substrate fabric (Figure 2).

### 2.3. Characterization

#### 2.3.1. Morphology

Zeiss Xradia 510 Versa 3D X-ray microscopes (XRM, (Carl Zeiss Microscopy Deutschland GmbH, Oberkochen, Germany) were used to take the 3D images and extract the volume of the area of the conductive yarn according to the shape of each electrode. The measured voltage and power were 50 kV and 4 W, respectively, and the resolution was 22 μm. The number of measured images was 1601 per sample, and the exposure time was five seconds.

#### 2.3.2. Digital Image Correlation (DIC)

**Figure 3:**
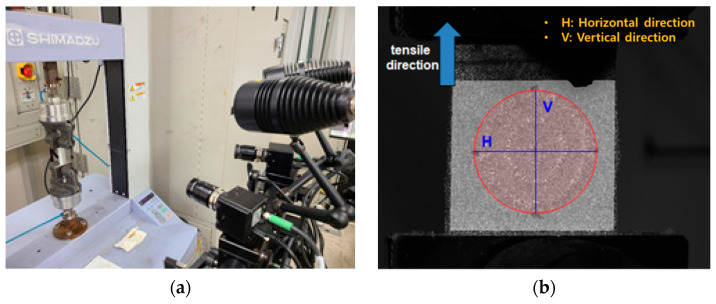
Measurement of the digital image correlation: (**a**) tensile tester and DIC system and (**b**) image of sample that positioned on tensile tester.

The deformation of the embroidery electrode shape was quantified according to the movement when applied as smart wear by image processing to measure the strain according to the tensile test. The tensile test was performed in a uniaxial direction using a tensile tester (Autograph AGS-X, Shimadzu Co., Ltd., Kyoto, Japan). At that time, ARAMIS from OMAGOM, a DIC system, was used to investigate the strain, as shown in Figure 3. Two samples were prepared for the test: one of 50 mm width × 150 mm length with a single electrode and the other with 50 mm width × 200 mm length with a bipolar electrode. The length of the horizontal and vertical directions and area of the samples elongated at 0%, 10%, 20%, and 30% by the tensile test were measured using the DIC. The condition of data acquisition frequency, subset size, and point distance were 2 Hz, 25 pixels, and 14 pixels, respectively. Through the test, the change in length of the horizontal and vertical directions according to the tensile strength and area were analyzed.

#### 2.3.3. Skin-Electrode Impedance

A sample with two embroidery electrodes located in parallel on the leg sleeve was used to compare the skin-impedance value according to the shape of the embroidery electrode. After wearing a leg sleeve so that the embroidery electrode could contact human skin, a disposable Ag/AgCl electrode was attached to the back of the embroidery electrode to measure the impedance. The distance between these two electrodes was kept at 40 mm, and the impedance was tested using an impedance analyzer (ZIVE P2 ELECTROCHEMICAL WORKSTATION, Won-A tech. Co., Ltd., Seoul, Korea). The skin-electrode impedance was measured with an AC sinusoidal signal at the 1.0 to 1000 kHz frequency range. For comparison according to the electrode type, the skin-electrode impedance value at 100 kHz was analyzed. During the experiment, no pre-treatments of the skin, such as shaving or exfoliation, were carried out separately because people would not implement skin preparations when wearing sEMG suits in practical use. All measurements were performed on the same day on the same subject.

#### 2.3.4. sEMG Measurement and Data Processing

The sEMG signals of various embroidery electrodes were evaluated by analyzing the EMG signal characteristics during knee extension. According to a previous study [28], bipolar EMG recordings were obtained using embroidery electrodes on the rectus femoris, which is located on the anterior thigh and is involved in knee extension and hip flexion. Before the test, no skin preparation was performed for the same reason mentioned above. Both electrodes were positioned on the midpoint of the muscle belly for the rectus femoris. A reference electrode, pre-gelled self-adhesive Ag/AgCl electrodes (Kendall LTP, Covidien, MA, USA), was placed over the head of the fibula, the electrically neutral bony prominence. The recordings were amplified and filtered (20–500 Hz) in analog (MP160, BIOPAC Systems Inc., Goleta, CA, USA). The data were full-wave rectified and averaged with a 100 ms time constant to draw the amplitude of the signals. The entire data processing of sEMG was performed using AcqKnowledge 5.0.1 Software (BIOPAC Systems Inc., Goleta, CA, USA).

**Figure 4:**
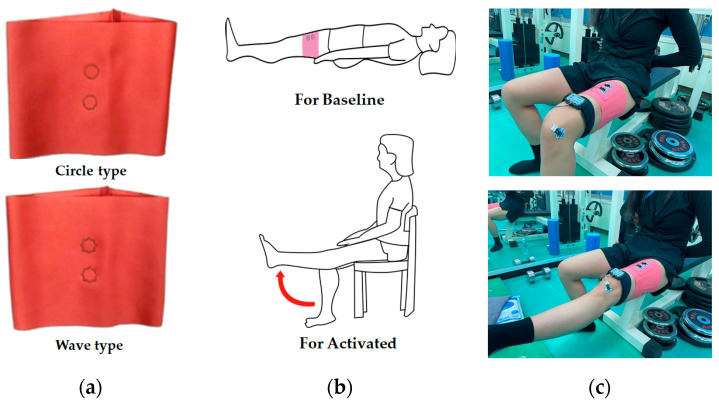
Images for measurement for sEMG signal: (**a**) actual leg sleeve; (**b**) testing posture; (**c**) actual sEMG signal testing.

Figure 4 presents an actual sample, the testing posture, and a measurement image during the sEMG measurement. Before testing a muscle contraction, the baseline EMG signals were collected in the supine position for 10 s to measure the baseline electrode noise (Figure 4b). With the subject in the seated position, a single-joint knee extension was conducted to induce muscle contraction signals. The subject extended the knee until the lower leg became parallel to the floor. Testing consisted of five consecutive trials for knee extension and knee flexion, and each phase lasted for five seconds. In this study, individual differences in muscle activities were not considered because the study was performed on only one subject. The entire experiment was conducted at room temperature under 60% RH. The selected test action was conducted five times to record the sEMG signal from one subject during intermittent muscle contraction measured using various electrodes. Three contractions among five, except the first and last trials, were used to calculate the average activated EMG for comparison.

**Figure 5:**
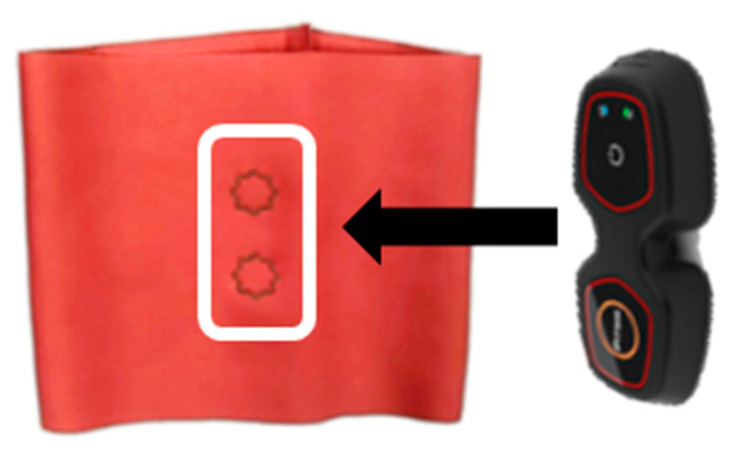
Images for measuring the sEMG signal using a wearable device: Measured by attaching a wearable device on top of the embroidery electrode in the white box.

Figure 5 shows the images for measuring the sEMG signal using a wearable device. This study examined whether the sEMG signal was obtained using a commercial wearable device and mobile application to confirm the applicability of the embroidery electrode for sportswear. The metal electrode part of a commercial wearable device (Fitsig, Roem system, Co., Ltd., Daejeon, Korea) was attached to the embroidery electrode part on the surface of the leg sleeve, and the embroidery electrode on the backside collected the signal of human skin. The sEMG signal collection method is the same as Biopac; three contractions among five, except for the first and last trials, were analyzed. The sEMG signal was compared according to the shape of each electrode and compared with the signal obtained from the Biopac.

## 3. Results and Discussion

### 3.1. Morphology of the Embroidery-Based Textile Electrodes

Table 2 lists the 3D images and area of conductive yarn of the embroidery-based textile electrodes. In this study, to manufacture an embroidery-based textile electrode for application to sportswear capable of collecting electromyography bio-signals, the electrode shape was developed into a circle-type, which is the same shape as the existing gel electrode, and a wave-type, which is a shape that can be stretchable [29,30]. In this case, the design was subdivided into the full-fill type, 3-line type, and 1-line type. As shown in Table 2, embroidered electrodes were manufactured based on the design. The fabricated electrodes were compared by deriving the area and total length of the conductive yarn through digital and 3D images. The digital image showed that the embroidery-based textile electrode was manufactured in the same way as the designed shape. According to the XRM data, a 3D image of the sample and the area of the conductive material were obtained. The wave-type electrodes had a larger amount of conductive yarn and area than the circle-type electrodes. The usage and area of the conductive yarns decreased with decreasing density. Previous studies reported that the size of the electrode affects signal collection [21,28]. These results appear to affect where the conductive material exists when EMG signals are collected with electrodes of the same size in the same muscle area. Accordingly, in this study, the amount and area of conductive yarn and the shape were analyzed according to the shape of the electrode, and a shape capable of detecting the same EMG signal was derived while using a smaller amount of conductive yarn than the Cf and Wf produced by filling the entire area.

### 3.2. Digital Image Correlation System Analysis of the Embroidery-Based Textile Electrodes

Table 3 and Table 4 list the results of digital image correlation analysis of the single-type and double-type embroidery-based textile electrodes according to the tensile change. Generally, elasticity must be considered when wearing clothes. In this study, for application to sportswear that requires more elasticity than basic clothing, the shape change and area change of the textile-based embroidery electrodes were examined when stretched by 10%, 20%, and 30% for each electrode.

Table 3 shows the change in the shape of a single electrode through DIC image analysis. The part expressed in blue and red indicates low strain (%) and high strain (%), respectively. The part expressed in blue is the part with the textile-based embroidery electrodes and represents less strain than the substrate fabric part. A comparison of Cf and Wf showed that both electrodes were relatively unchanged at 10% strain. As the strain increased, the shape of the electrode was also stretched in the tensile direction, and the width decreased in the transverse direction. At this time, when Cf and Wf of 30% strain were observed, the width of the Wf was smaller than that of Cf. This result was similar to the results of C3 and W3, C1 and W1. In addition, a comparison of the fill, 3 lines, and 1 line electrodes confirmed that the wave-type is morphologically stable despite the decrease in the area where the conduction is present. In the case of the circle-type, however, the strain in the tensile direction increased with decreasing area embroidered with the conductive yarn. This is because the wave type with a curve has more areas that can be stretched during tension than the linear circle. Thus, it was confirmed that the morphologically stable electrode is less deformed than the circle-type electrode [29,30]. Furthermore, compared to fill, 3 lines, and 1 line, the strain also increased because the area of the substrate fabric that can be stretched increases with decreasing area embroidered with the conductive yarn.

A bipolar electrode is required to measure the EMG signal [12,13]. Accordingly, Table 4 lists DIC images of the shape change by the strain after positioning the bipolar electrode with the IDE set to 40 mm as the distance between the electrodes based on a previous study that measured the signal in the rectus femoris region. First, when Cf and Wf were compared, as bipolar electrodes were stretched to 30% strain, the strain of the stretchable substrate fabric between the electrodes increased gradually to show the maximum strain, but the electrodes were not deformed. In addition, when elongated to 30%, the transverse direction of the substrate fabric exhibited a necking phenomenon that generally appears in materials due to tensile changes. C3 and W3 also showed similar trends to Cf and Wf. In the case of the textile-based embroidery electrode, there was no significant change with tensile changes up to 30% strain, but only the substrate fabric between the electrodes was affected. In the case of the necking phenomenon of the substrate fabric, the shrinkage was less than that of Cf or Wf through the DIC image. C3 and W3 were less affected by the presence of the elastic substrate fabric between the three line-type areas filled with conductive yarns than Cf or Wf, which were filled with stiff conductive yarns. C1 and W1 showed some different aspects than the above two electrodes. C1 or W1 were also elongated as the strain was increased to 30%, indicating that the length in the tensile direction was elongated. The strain was also affected by the inner substrate fabric. Hence, the stress was not concentrated on the substrate fabric between the two electrodes, and it was elongated overall; necking was hardly observed.

The size or shape of the electrode affects the noise when collecting EMG signals from a target muscle [21]. Accordingly, the shape of the electrode is maintained as much as possible depending on the strain as an important point of signal collection. DIC image analysis confirmed that the wave-type deformation was more stable than the circle-type shape through the single electrode shape analysis. In addition, the tensile strain was small when the 3-line shape was employed through the shape analysis of bipolar electrodes for signal collection.

**Figure 6:**
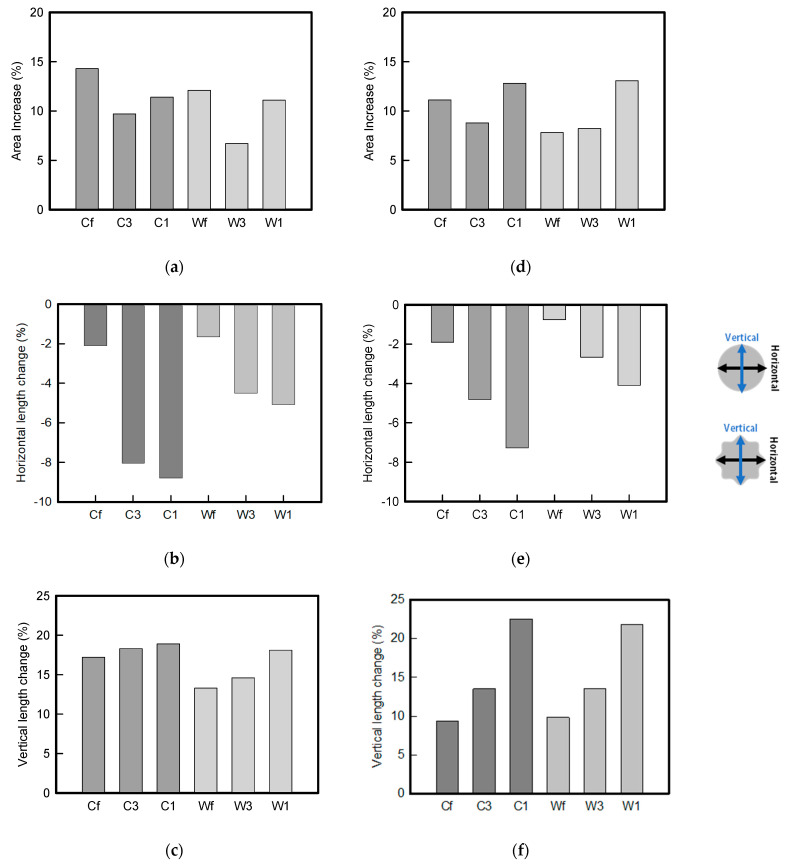
DIC system analysis results of circle- and wave-type embroidery-based textile electrodes for elongation at 30%; (**a**) area increase; (**b**) horizontal length change; (**c**) vertical length change in a single electrode; (**d**) area increase; (**e**) horizontal length change; (**f**) vertical length change of a bipolar electrode.

Figure 6 shows the analysis results through the DIC image for elongation at 30% as a graph. Figure 6 also indicates the area increase and length change of horizontal and vertical direction for circle and wave type of embroidery-based textile electrodes. A comparison of the area of a single electrode showed that the area increase in Cf, C3, and C1 was 14.3%, 9.7%, and 11.4%, respectively, and the area increase in Wf, W3, and W1 was 12.1%, 6.7%, and 11.1%, respectively (Figure 6a). Both the circle type and wave type were indicated in the order of 3 lines < fill < 1 line.

As shown in Figure 6b,c, which is the DIC image analysis result, the length change in the vertical direction, which is the same direction as the tensile direction, shows a positive value as it lengthens in the lengthwise direction. By contrast, the length change in the horizontal direction, which is the opposite direction, shows a necking phenomenon and a negative value. An analysis of the length change of circle-type and wave-type confirmed that both horizontal and vertical length changes of the wave-type were smaller than that of the circle-type. A comparison according to the amount of conductive yarn used showed that the deformation was large in the order of fill < 3 lines < 1 line. In terms of the length change, the smallest change was observed in the fill-type, but three lines were less deformed in the area increase, confirming that the overall shape deformation was maintained more stably in the 3 lines.

This phenomenon was also observed with the bipolar electrode. As shown in Figure 6d, the total area change was fill < 3 lines < 1 line. The horizontal and vertical length changes were reduced by approximately 1/2 compared to the single electrode (Figure 6e,f). There was less shape deformation than in the case of a single electrode because of the substrate fabric between the electrodes. At the two shape changes, the wave-type deformation was less than that of the single electrode. In addition, fill < 3 lines < 1 line according to the number of lines. As mentioned above, the length change at the bipolar electrode was the smallest when the fill-type was done, but 3 lines were less deformed in the area increase. Three lines will be more stable in the overall shape deformation when applied as EMG fitness smart wear later.

### 3.3. Skin-Electrode Impedance Analysis of the Embroidery Based Textile Electrodes

**Figure 7:**
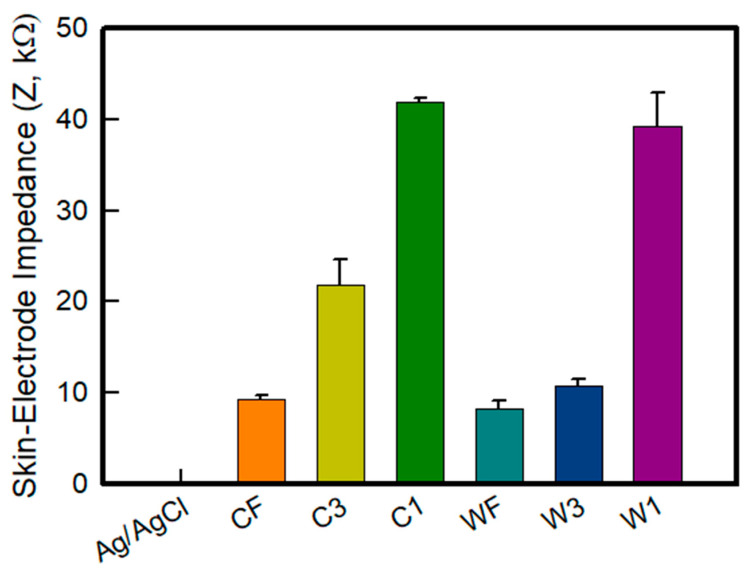
Skin-electrode impedance of embroidery-based textile electrodes with various types.

Generally, a higher skin-contact impedance leads to an increased impedance mismatch that reduces common-mode rejection and, hence the SNR (signal to noise ratio) [31,32]. Therefore, it is important to decrease and control the impedance to manufacture an electrode for bio-signals. Figure 7 shows a graph of the skin-electrode impedance of the embroidery-based textile electrodes of various types. The skin-impedance was measured to confirm each sample type through the skin-impedance measurement performance of the electrode itself. The impedance at 100 kHz of the Ag/AgCl electrode, Cf, C3, C1, Wf, W3, and W1 was measured to be 0.210 ± 0.005 kΩ, 9.358 ± 0.372 kΩ, 21.892 ± 2.766 kΩ, 41.845 ± 0.480 kΩ, 8.247 ± 0.967 kΩ, 10.789 ± 0.775 kΩ, and 39.226 ± 3.727 kΩ, respectively. The embroidery electrode showed an impedance value that was at least 40 times higher than the Ag/AgCl electrode. It is confirmed that the Ag/AgCl electrode showed a relatively low value because it was measured in direct contact with the skin. According to the electrode type, the skin-impedance analysis revealed the wave-type to have a relatively higher impedance than the circle-type. According to the number of lines of electrodes, the trend of fill < 3 lines < 1 line was followed. A previous study analyzing the effect of the electrode area on the impedance reported that the impedance decreased with increasing cross-sectional area, indicating an inversely proportional relationship [22,32]. This result was attributed to the area in contact with the skin also increasing due to the increase in the density and area of the conductive material in the electrode of the same size when the amount of conductive yarn was increased. In addition, in relation to the DIC analyzed above, it is confirmed that the wave-type skin-electrode impedance of the electrode, which is stable even in tension, is excellent. Accordingly, it was found that the skin-electrode impedance decreases as the amount of conductive yarn that can be collected within the same area increases, which will also affect the sEMG signal collection.

### 3.4. sEMG Signal and Average Rectified sEMG of the Embroidery-Based Textile Electrodes

Based on a previous study [28], to investigate the sEMG, the signal of the rectus femoris was acquired by repeating the knee flexion-extension process five times while wearing a leg sleeve containing the embroidery electrodes. The sEMG signal was obtained for each electrode by repeatedly measuring five seconds of release-and-rest periods following each flexion.

**Figure 8:**
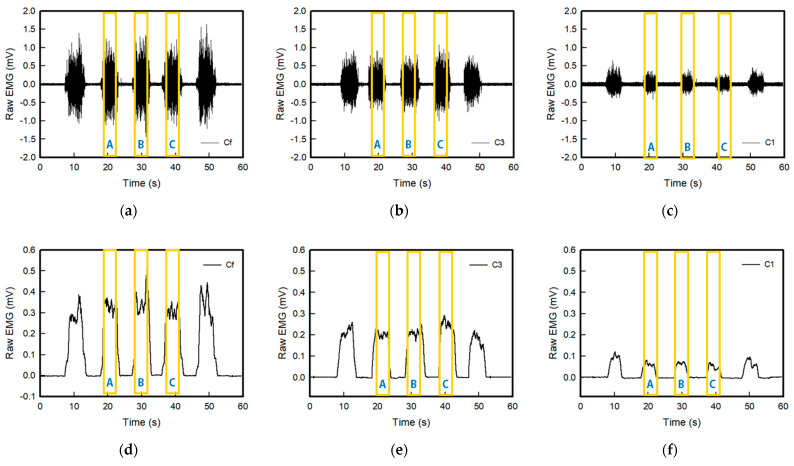
Graph of raw sEMG signals obtained via (**a**) Cf, (**b**) C3, and (**c**) C1 and filtered (20–500 Hz) in analog and full-wave of the sEMG signal of (**d**) Cf, (**e**) C3, and (**f**) C1, respectively; Here, the values of sections A, B, and C in the yellow box were used for EMG signal measurement and analysis.

**Figure 9:**
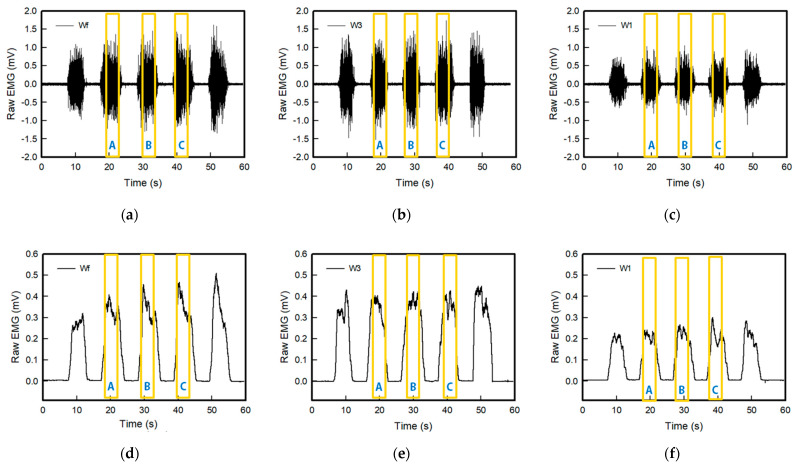
Graph of raw sEMG signals obtained via (**a**) Wf, (**b**) W3, and (**c**) W1 and filtered (20–500 Hz) in analog and full-wave of the sEMG signal of (**d**) Wf, (**e**) W3, and (**f**) W1, respectively; Here, the values of sections A, B, and C in the yellow box were used for EMG signal measurement and analysis.

Figure 8 and Figure 9 show the raw sEMG signals and full wave of the sEMG signal of the embroidery-based textile electrodes. All embroidery-based textile electrodes detected the sEMG signal of the muscle activation. In addition, the sEMG signal changed according to the shape and area of the electrode. The strength of the detected signal of the wave-type signal was larger than the circle-type, and it tended to increase with an increasing number of lines: 1 line < 3 lines < Fill. In the case of the wave type, the strengths of the EMG signal of Wf and W3 were similar. Moreover, the signal collection of W3 was more stable than other electrodes. In addition, the signal strength of C1 and W1 was relatively lower than that of other electrodes. Therefore, sufficient signal strength was not provided, which was affected by the shape of the electrode analyzed above and the embroidered area of the conductive yarn. This is because the wave-type is more stable to shape changes in tension than the circle-type; the signal strength appears to be greater. In addition, according to the amount and area of the conductive yarn, the electrode with a large area that can contact the skin is the fill type. On the other hand, the signal strength of W3 was highest because the 3-line electrode type showed little change in the shape of the electrode by elongation.

**Figure 10:**
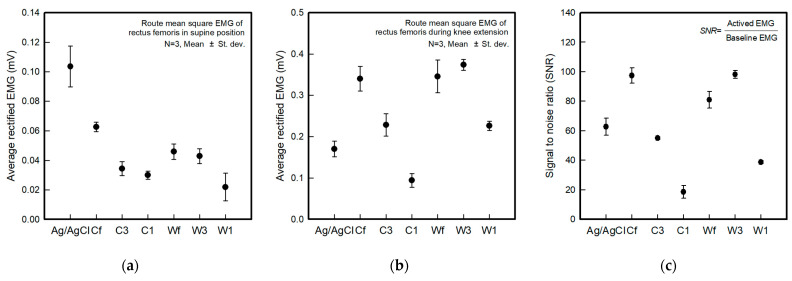
Average rectified sEMG of the baseline during muscle activation for different electrode types. (**a**) Baseline electrode noise; (**b**) sEMG amplitude during muscle contractions exerted by knee extension; (**c**) signal-to-noise ratio (SNR).

Figure 10 shows the average rectified sEMG of the baseline before and after muscle activation and the SNR for the different electrode types. The parameters for ambient noise were calculated using the muscle signal and SNR analysis, and the samples for three seconds were taken from the signals at the raw EMG.

The baseline electrode noise was different according to the shape and embroidered area of the electrodes (Figure 10a). The baseline noise of the Ag/AgCl electrode was 0.104 ± 0.014 mV, and the Cf, C3, C1, Wf, W3, and W1 electrodes were 0.063 ± 0.003 mV, 0.034 ± 0.003 mV, 0.030 ± 0.005 mV, 0.046 ± 0.005 mV, 0.043 ± 0.005 mV, and 0.022 ± 0.009 mV, respectively. The textile-based embroidery electrodes had lower baseline electrode noise levels than the Ag/AgCl electrodes. Hence, the textile-based embroidery electrodes can stably collect signals when there is no muscle movement. The results of the EMG signals during muscle contraction showed different values depending on the type of textile-based embroidery electrodes (Figure 10b). The Ag/AgCl electrode was 0.170 ± 0.019 mV, and the Cf, C3, C1, Wf, W3, and W1 electrodes were 0.340 ± 0.030 mV, 0.229 ± 0.027 mV, 0.094 ± 0.017 mV, 0.346 ± 0.040 mV, 0.374 ± 0.013 mV, and 0.226 ± 0.011 mV, respectively. The remaining electrodes, except for C1, exhibited higher EMG signal performance than the reference electrode, Ag/AgCl. In terms of the electrode types, the wave-type textile-based embroidery electrodes showed superior EMG signal-detected performance compared to the circle-types. Furthermore, when analyzed according to the embroidered area, 1-line electrodes showed a relatively low EMG signal strength, and 3-line and fill electrodes were similar. In particular, W3 showed better performance than the fill types Cf and Wf. As analyzed previously in the DIC image, the EMG signal strength according to muscle contraction was also high because W3, which is morphologically stable according to the change in elongation, can maintain the electrode shape by operation.

The SNR was calculated as the ratio of active EMG to the baseline EMG. The SNR showed different results depending on the shape of the electrode and the area embroidered with the conductive yarn (Figure 10c). The Ag/AgCl electrode was 62.8 ± 5.9, and the Cf, C3, C1, Wf, W3, and W1 were 97,4 ± 5.3 mV, 54.9 ± 0.9 mV, 18.5 ± 4.3 mV, 81.0 ± 5.5 mV, 98.1 ± 2.7 mV, and 38.8 ± 1.0 mV, respectively. A comparison with the SNR of the reference electrode showed similar or higher values except for C1 and W1, which are 1-line types. In addition, compared to previous studies related to fabricating textile-based dry electrodes, including the embroidery technique, the baseline noise of the current results was lower, and the SNR was better [21,27,28]. Finally, the W3 electrode showed the highest SNR among all the electrodes, confirming that a more stable electrode shape has a more stable signal collection.

**Figure 11:**
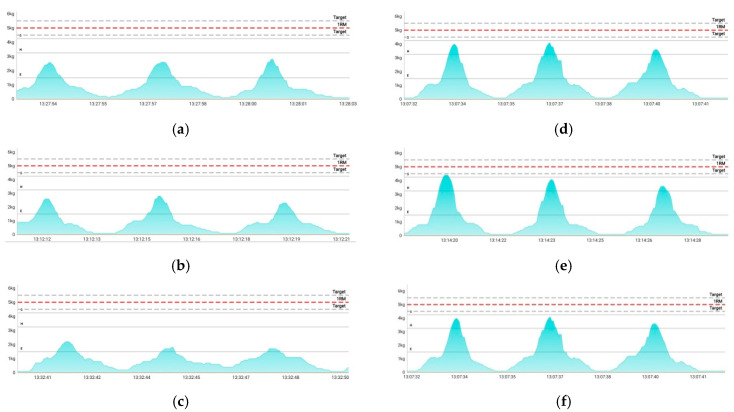
Images of collected sEMG signals by different types of embroidery-based textile electrodes with wearable devices. (**a**) Cf; (**b**) C3; (**c**) C1; (**d**) Wf; (**e**) W3; (**f**) W1.

Figure 11 presents images of the collected sEMG signals according to different types of embroidery-based textile electrodes with wearable devices to confirm the applicability of the textile-based embroidery electrode to smart electromyography wear for fitness. The devices and applications used in this study are products for the public. Accordingly, it is indicated that the measured voltage is converted into a perceived weight to make it easier to recognize the EMG recognition. As shown in Figure 10, after wearing all the samples that embedded the textile-based embroidery electrode, the EMG signals could be collected by combining a wearable device. In addition, the EMG signal strength showed the same tendency as the SNR analyzed previously, confirming that the commercialization of smart electromyography clothing will be possible in the future.

## 4. Conclusions

Textile-based electrodes were fabricated for sEMG detection in EMG module-combined smart wear. The electrodes were manufactured with different shapes and conductive areas using embroidery techniques, and their sEMG performance was analyzed.

For more accurate EMG measurements, the morphological changes were analyzed according to the electrode shape and strain because the shape of the electrode must be maintained according to wearing and movement. The area where the wave-type electrode could be stretched was larger than that of the circle-type electrode, so it was stable in shape deformation according to the increase in strain. An analysis of the shape deformation of a sample prepared with a bipolar electrode for sEMG measurements confirmed that the shape of the electrode was maintained while necking between the two electrodes was reduced when the 3-line electrode was manufactured. Skin-electrode impedance analysis confirmed that as the embroidered area with conductive yarn increased, the area in contact with the skin increased, and the impedance decreased. As a result of analyzing the EMG signal strength according to the shape of the electrode and the embroidered area, the EMG signal strength was superior to that of the Ag/AgCl electrode, which is the reference electrode, except for the 1-line type electrodes. In addition, the wave-type sample showed a higher EMG signal strength than the circle-type. In particular, the W3 electrode showed better performance than the fill-type Cf and Wf. The EMG signal detection performance was excellent because the electrode shape was maintained relatively well according to wearing and operation. These performances confirmed that the device could be operated without noise, even with a commercial device.

Accordingly, when manufacturing the embroidery electrode for EMG signal acquisition, it was confirmed that it was morphologically stable up to 20% tensile when manufactured with a wave-type of 3 lines. It was affected by EMG signal acquirement; W3 confirmed that it is possible to obtain stable EMG signal acquisition with less noise. Therefore, the manufacture of smart clothing based on electromyography using the embroidered electrodes is expected.

## Figures and Tables

**Table 1:** Sample code and images of the embroidery electrode used in this study.

Type	Sample Code	Number of Lines	Designed Image	Sample Image
Circle	Cf	Fill	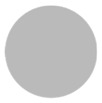	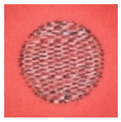
	C3	3	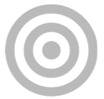	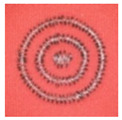
	C1	1	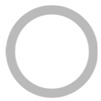	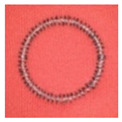
Wave	Wf	Fill	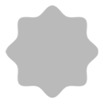	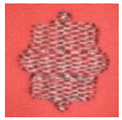
	W3	3	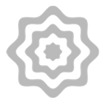	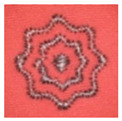
	W1	1	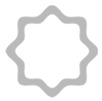	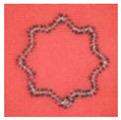

**Table 2:** 3D image and area of conductive yarn of embroidery-based textile electrodes.

	Sample Code					
	Cf	C3	C1	Wf	W3	W1
3D image	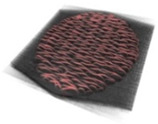	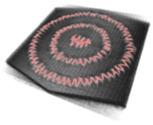	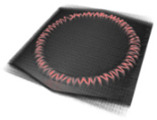	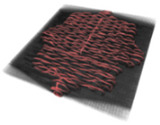	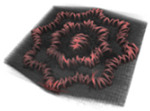	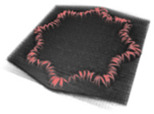
Area of conductive yarn	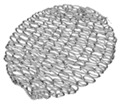	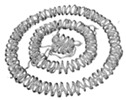	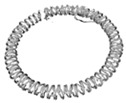	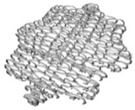	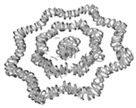	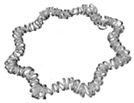
Total length (mm)	812.0	565.0	360.0	822.0	693.0	419.0
Total area (mm^2^)	32.5	28.8	16.7	36.6	24.5	22.3

**Table 3:** DIC images of the single-type embroidery-based textile electrode by tensile change.

	10%		20%		30%	
	Circle	Wave	Circle	Wave	Circle	Wave
Fill	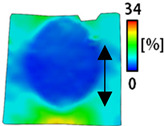	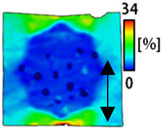	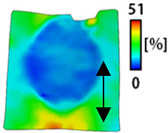	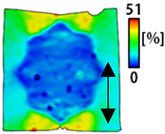	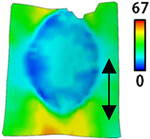	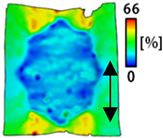
3	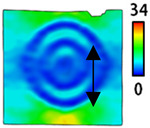	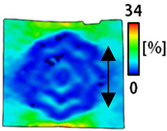	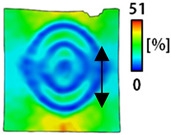	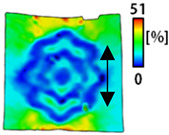	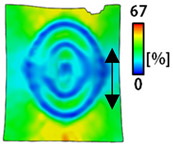	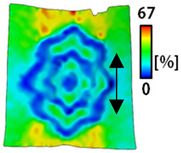
1	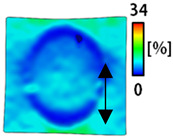	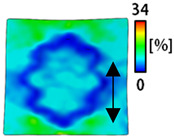	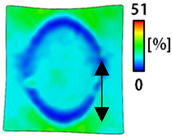	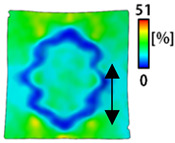	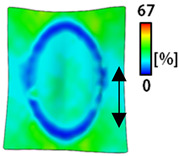	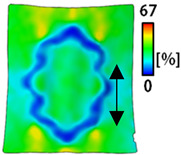

**Table 4:** DIC images of the bipolar embroidery-based textile electrodes by tensile change.

	10%		20%		30%	
	Circle	Wave	Circle	Wave	Circle	Wave
Fill	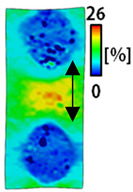	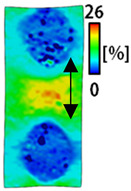	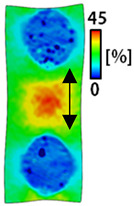	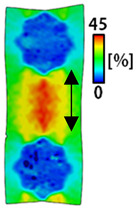	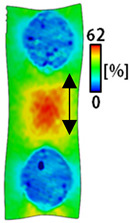	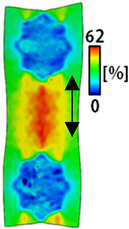
3	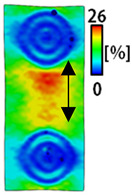	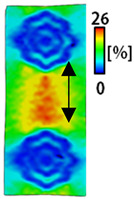	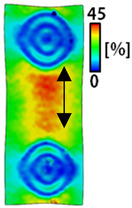	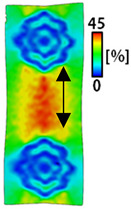	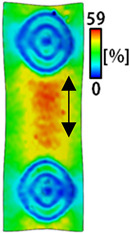	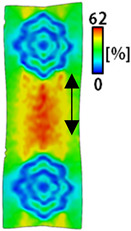
1	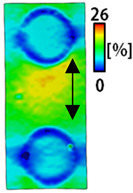	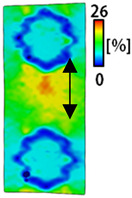	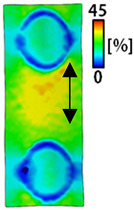	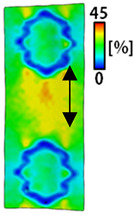	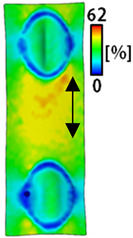	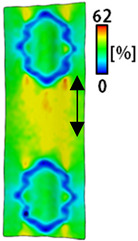

## Data Availability

The data presented in this study are available in this article.
